# The Lilliput Effect in Colonial Organisms: Cheilostome Bryozoans at the Cretaceous–Paleogene Mass Extinction

**DOI:** 10.1371/journal.pone.0087048

**Published:** 2014-02-05

**Authors:** Caroline E. Sogot, Elizabeth M. Harper, Paul D. Taylor

**Affiliations:** 1 Department of Earth Sciences, University of Cambridge, Cambridge, United Kingdom; 2 Department of Earth Sciences, Natural History Museum, London, United Kingdom; University of Birmingham, United Kingdom

## Abstract

Consistent trends towards decreasing body size in the aftermath of mass extinctions – Lilliput effects – imply a predictable response among unitary animals to these events. The occurrence of Lilliput effects has yet to be widely tested in colonial organisms, which are of particular interest as size change may potentially occur at the two hierarchical levels of the colony and the individual zooids. Bryozoans are particularly useful organisms in which to study colonial size response as they have well-defined zooids. Additionally, a number of analyses of present-day bryozoans have shown that zooid size reflects local environmental conditions, most notably seawater temperature and possibly also food supply. Following the hypothesised decline in primary productivity at the Cretaceous–Paleogene (K–Pg) mass extinction, it is predicted that bryozoan zooid size should decline in the early Paleogene, resulting in a Lilliput effect. To test this prediction, zooid size was compared across the K–Pg boundary at the assemblage level and also within 4 surviving genera. Analysis of 59 bryozoan species from assemblages on either side of the K–Pg boundary showed no significant change in zooid length. Zooid size was also measured in 98 Maastrichtian colonies and 162 Danian colonies belonging to four congeneric species. Only one of these genera showed a significant size decrease across the K–Pg boundary, the other three maintaining constant zooidal lengths, widths and areas. Additionally, the sizes of 210 Maastrichtian colonies and 163 Danian colonies did not show consistent size decrease across the K–Pg boundary in these same species, although maximum colony size did decline in three out of four genera. Furthermore, this lack of consistent size change is uniform between two distinct biogeographical regions, Denmark and the southeastern USA.

## Introduction

The Lilliput effect [1] describes a decrease in body size following mass extinctions and is often thought to reflect an ecophenotypic response to environmental changes at these times. If this phenomenon occurs in all taxa and across all mass extinctions, it means that organisms have reacted predictably to these events, regardless of their proximate cause [2]. Such predictability has the potential to assist our understanding of future responses by organisms to contemporary ecological disturbances [3]. Recent studies, however, have questioned the ubiquity of the Lilliput effect [4]–[7], prompting discussion of its importance.

The original definition of the ‘Lilliput effect’ originated from the observation of diminutive colonies among some graptolite species that survived the Late Silurian biotic crises [1]. The term has become modified over time; it is often used in a more general sense for any examples of small-sized, post-extinction organisms, as opposed to size change within lineages crossing mass extinctions [8]–[9]. As such, it is unclear how often the Lilliput effect, in terms of its original more restricted definition, really occurs [10]. Within-lineage size decrease has been studied less often as it ideally demands a reliable phylogeny [3]. Although patterns of within-lineage size change have been observed to be more variable than those reported for higher taxa [8], a Lilliput effect has been reported to occur in some instances at lower taxonomic levels (Table 1).

**Table 1:** Summary of within-lineage size trends across mass extinction intervals in invertebrate species or genera.

Size trends	Taxa		Mass extinction	Reference
**Size decrease**	Graptolites	*Pristiograptus dubius parvus* *	Upper Silurian	[1]
		*Pristiograptus dubius tumescens* *		
		*Saetograptus leintwardinensis* *		
	Brachiopods	?*Tethyochonetes* sp.		[54] a
		*Paryphella orbicularis*		
		*‘Lingula’* *		[9]
	Gastropod	*Bellerophon* *		
	Bivalve	*Pseuodmytiloides* *	Pliensbachian– Toarcian	[50] b
	Ammonite	*Dactylioceras* *		
	Brachiopods	*Deliella* *		
		*Strophomena* (and *Katastrophomena*)*	End-Ordovician	[8]
		*Skenidioides* *		
		*Coolinia* *		
**No change**	Belemnites	*Passaloteuthis milleri*	Pliensbachian– Toarcian	[55] b
		*Passaloteuthis bisulcata*		
**Initial decrease, then increase**	Brachiopods	*Paracraniops*	End-Ordovician	[8]
		*Brevilamnulella*		
**Size increase**	Brachiopods	*Triplesia*	End-Ordovician	[8]
		*Eospirifer*		

Only taxa that cross the extinction boundaries are considered.

* described in the original reference as a ‘Lilliput effect’.

a Other brachiopod species analysed in this study show varying trends prior to the Permo–Triassic boundary: two species decrease and two increase in size prior to the boundary. However, only the species listed in the above table cross over the Permo-Triassic boundary itself.

b Other taxa show variable size trends in this study, but do not cross the boundary itself. Those that do cross the boundary might also show variable size trends leading up to or beyond the boundary, e.g. *Passaloteuthis bisulcata* significant increases in size at the start of the extinction interval.

There are numerous Lilliput analyses for an array of unitary (solitary) organisms but very few for colonial organisms [10], despite the original concept being based on graptolites [1]. Colonial organisms are important components of modern benthic ecosystems (e.g. reef corals) and their size response has potential implications for other taxa dependent upon them. Understanding such reactions to changing environments is therefore of particular importance at the present day. Size changes documented for colonial organisms are of further interest as they can occur at two hierarchical levels: the colony and its constituent individual modules (zooids). Colony size reduction could potentially be the result of smaller zooids and/or fewer component zooids [1]. Paralleling solitary organisms, it might also be expected that the individual zooids would experience a reduction in size. However, zooid size change in colonial organisms has yet to be analysed across a mass extinction boundary.

The aim of this study is to gain new perspectives on the Lilliput effect by investigating changes across the Cretaceous–Paleogene (K–Pg) mass extinction in bryozoan size at the two hierarchical levels of the colony and the zooid. Cheilostome bryozoans lend themselves particularly well to studies of size change as their box-like zooids retain a fixed size after they are budded [11], and the perimeters of the typically sheet-like encrusting colonies are well defined, allowing precise measurement of colony area.

The K–Pg event has long been associated with a significant decline in primary productivity [12], which is considered a key driver of the Lilliput effect [2]. It is therefore hypothesised that survivors of the K–Pg mass extinction should exhibit smaller body size than their pre-extinction relatives [13]–[17]. In the case of bryozoans, this post-extinction size decrease can be expected at both the colony- and the zooid-level. Decreases in zooid size and colony size have been observed in cheilostome bryozoans during the latest Maastrichtian in Denmark and are thought to represent unstable and unfavourable environmental conditions, with low planktonic productivity, prior to the K–Pg boundary [18]. The study here extends this analysis across the K–Pg boundary to establish whether size reduction continued into the early Danian.

## Materials and Methods

### Geological Setting

No specific permissions were required for fieldwork, which was carried out on public land. Field studies did not involve endangered or protected species.

Cheilostome bryozoan size is assessed in two regions – southeastern USA and Denmark – to account for biogeographical variation. Although Maastrichtian bryozoan faunas occur in several regions, Paleocene faunas are much rarer [19], making it difficult to compare bryozoans across the K–Pg boundary beyond the two regions studied here [20].

**Figure 1:**
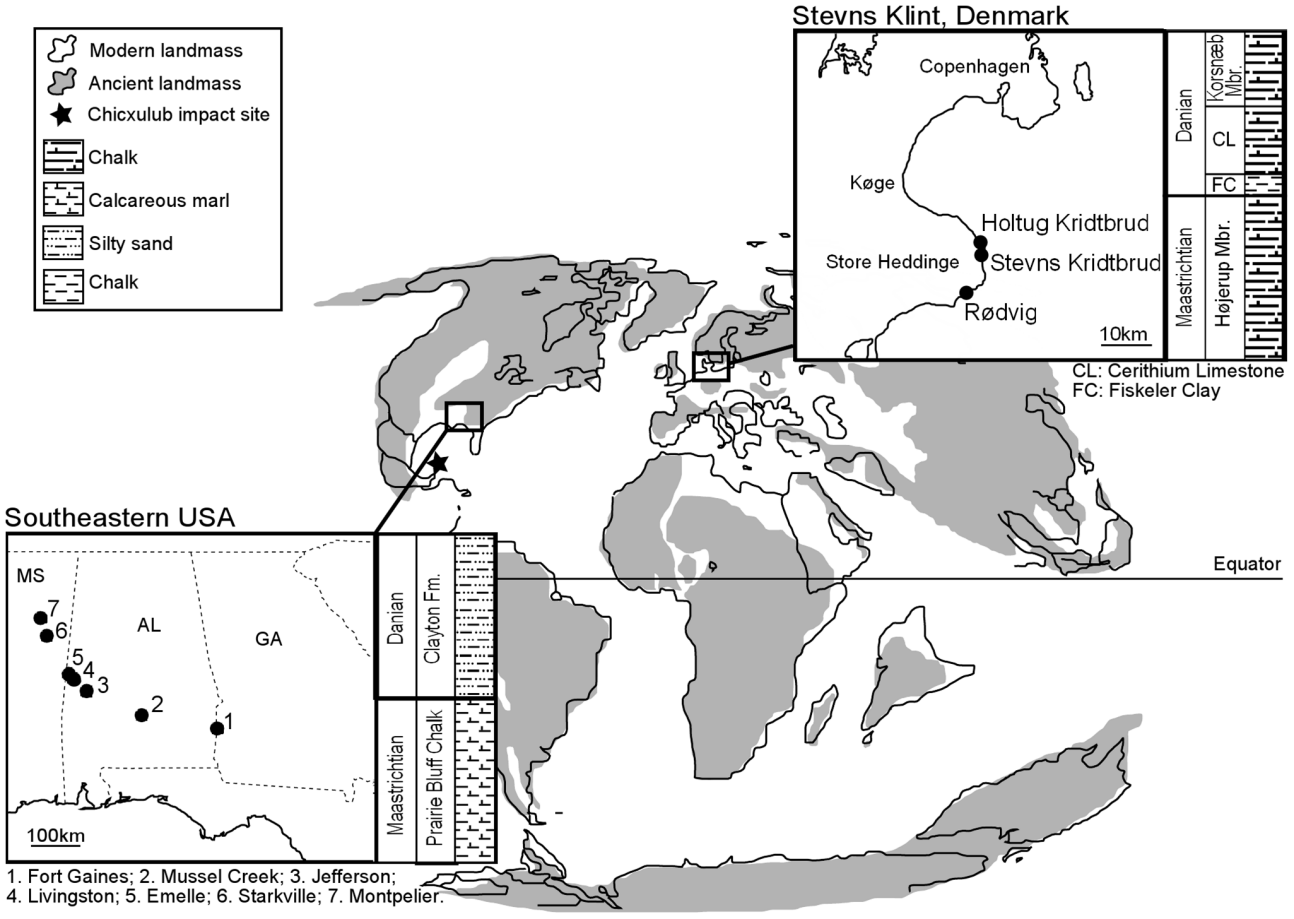
Locality maps and stratigraphical logs for the two study regions, Denmark and the southeastern USA. Stratigraphical formations and members are shown to highlight the units from which specimens were collected for analysis. Global palaeomap adapted from: http://scotese.com/K/t.htm.

In the southeastern USA, encrusting cheilostome specimens were collected from seven localities across Georgia, Alabama and Mississippi (Figure 1). Fieldwork was undertaken on public land and no protected or valuable specimens were collected. This area was a shallow marine shelf at a palaeolatitude of approximately 33°N in the Late Cretaceous and Early Paleocene [21]. Sediments represent deltaic and nearshore marine settings [22], and occur in mixed carbonate and siliclastic sequences [23]. Most of the bryozoans encrusted ‘oyster’ shells (*Exogyra*, *Pycnodonte,* etc.) and were collected from the Maastrichtian Prairie Bluff Chalk and the Danian Clayton Formation.

In Denmark, specimens were collected from three localities at Stevns Klint, 45 km south of Copenhagen (Figure 1). No specific permissions were required and no protected or valuable specimens were collected. Stevns Klint was situated at approximately 45°N in the Late Cretaceous and Early Paleocene [24]. The K–Pg sections here consist of virtually pure chalk, contrasting with the more siliciclastic sediments of the southeastern USA. At Stevns Klint, cheilostome colonies mostly encrust echinoid tests, particularly *Echinocorys*. Maastrichtian specimens are from the Højerup Member whereas Danian specimens are from the Paleogene Korsnæb Member overlying the intervening Danian Cerithium Limestone which contains few bryozoans.

Zooid size and colony size were measured in encrusting sheet-like cheilostome bryozoans. Zooid size changes were analysed at both the assemblage level and within surviving clades to provide two different comparisons of size trends across the K–Pg boundary.

### Within-genus colony size

**Figure 2:**
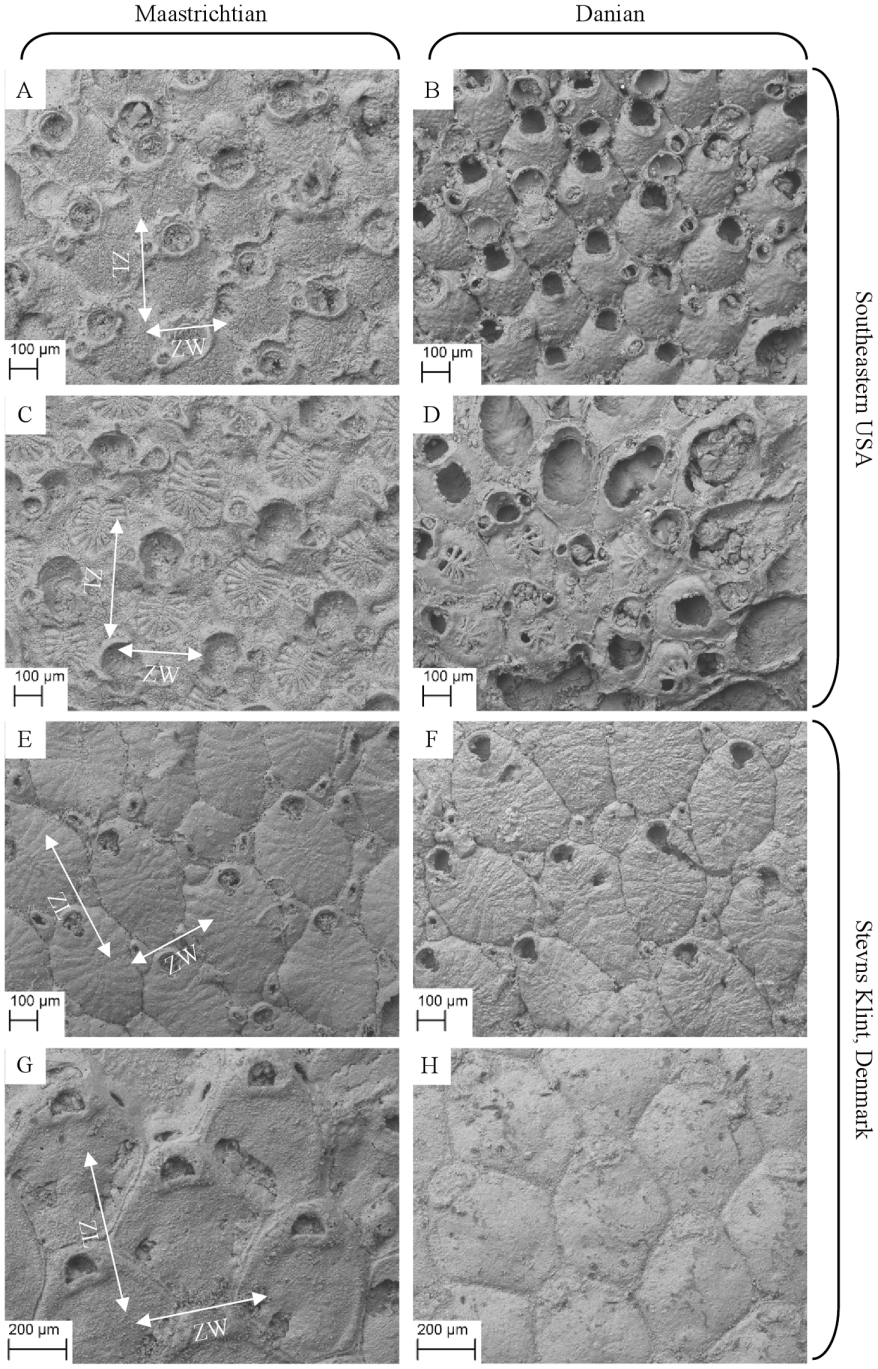
SEM images of species used in this study. Left-hand column shows Maastrichtian specimens, right-hand column Danian specimens. Rows are congeneric (or conspecific) pairs. Examples of maximum zooid length (ZL) and maximum zooid width (ZW) measurements are shown on the Maastrichtian specimens. A. *Balantiostoma nomas* (Shaw, 1967); B. *Balantiostoma midwayanica* (Canu and Bassler, 1920); C. *Tricephalopora larwoodi* (Shaw, 1967); D. *Tricephalopora levigatum* (Canu and Bassler, 1920); E. *Pliophloea subcornuta* (Berthelsen, 1962); F. *Pliophloea subcornuta*; G. *Stichomicropora* sp. 1; H. *Stichomicropora* sp. 2.

To minimise the potential confounding influence of phylogenetic differences, and in keeping with the original definition of the Lilliput effect, the sizes of closely related congeneric species were compared before and after the K–Pg boundary (Figure 2). Although some generic names traditionally used for these species in the Cretaceous and Paleocene species in the USA differ [25]–[26], the selected pairs of species from either side of the K–Pg boundary are here considered to be congeneric and, for *Pliophloea subcornuta*, conspecific. The lack of a phylogenetic study of these bryozoans, which would have identified sister groups, forced this selection of these taxa, which are based on morphological similarities.

Colony sizes were compared across the K–Pg boundary in two congeneric pairs from the USA and two from Denmark (Figure 2). Every colony of these species present in the collections was measured. This meant that colonies were sampled from multiple localities within each region. In total, the areas of 136 colonies from the USA (72 Maastrichtian, 64 Danian) and 237 colonies from Denmark (138 Maastrichtian, 99 Danian) were measured (Tables 2, 3).

**Table 2:** Number of (A) colonies measured, (B) zooids measured and (C) species, from which zooids were measured in the USA.

Formation	Prairie Bluff				Clayton			Total		
Locality	M.	L.	E.	J.	F. G.	M.C.	S.	Prairie Bluff	Clayton	Total
**A) Taxon**	**Number of colonies**									
*Balantiostoma*	-	4	6	17	-	38	16	27	54	81
*Tricephalopora*	-	15	2	28	-	2	8	45	10	55
**Total**								**72**	**64**	**136**
**B) Taxon**	**Number of zooids**									
*Balantiostoma*	30	40	-	-	50	40	-	70	90	160
*Tricephalopora*	60	50	-	-	20	10	-	110	30	140
**Total**								**180**	**120**	**300**
**C) All species**	**Number of species**									
**Total**								**20**	**2**	**22**

Locality abbreviations: M.  =  Montpelier; L.  =  Livingston; E  =  Emelle; J  =  Jefferson; F. G.  =  Fort Gaines; M. C.  =  Mussel Creek; S  =  Starkville.

**Table 3:** Number of (A) colonies measured, (B) zooids measured and (C) species, from which zooids were measured in Denmark.

Member	Højerup			Korsnæb			Total		
Locality	H.	S.	R.	H.	S.	R.	Højerup	Korsnæb	Total
**A) Taxon**	**Number of colonies**								
*Pliophloea subcornuta*	67	5	-	-	24	37	72	61	133
*Stichomicropora*	30	7	29	7	23	8	66	38	104
**Total**							**138**	**99**	**237**
**B) Taxon**	**Number of zooids**								
*Pliophloea subcornuta*	50	-	-	-	30	80	50	110	160
*Stichomicropora*	10	-	20	-	40	-	30	40	70
**Total**							**80**	**150**	**230**
**C) All species**	**Number of species**								
**Total**							**25**	**12**	**37**

Locality abbreviations: H.  =  Holtug Kridtbrud; S.  =  Stevns Kridtbrud; R.  =  Rødvig.

Photographs were taken of each colony and their areas were measured using ImageJ software (http://rsbweb.nih.gov/ij/). The mean, minimum and maximum colony area was determined for each species, before and after the K–Pg boundary. The coefficient of variation (CV) was also calculated by dividing the standard deviation of colony sizes for each species by the mean colony size.

The perimeters of encrusting cheilostome colonies are generally well defined. However, preservational losses make it impossible to measure the original sizes of all colonies as breakage of colony edges means that measured colony areas underestimate the true area. However, as there is no evidence that breakage differed systematically between Maastrichtian and Danian samples, this factor is believed not to bias the results.

The hierarchical nature of the dataset – colonies, localities and stratigraphical intervals – lends itself to nested ANOVA analysis [27]–[30]. A general linear model was used to conduct the analysis, as this is an ANOVA method that allows for non- matching sized data sets. This method allows the comparison of size at different levels: (1) variation between colonies to test for variation within species from the same environment and time; (2) variation between localities, to test for variation between colonies from a given stratigraphical horizon; and (3) variation between formations to evaluate variation across the K–Pg boundary. Nested ANOVA therefore allows assessment of whether variation is indeed between formations on opposite sides of the K–Pg boundary, removing these other potentially confounding factors. It also allows a comparison of the proportion of variation that each factor contributes overall, by calculating the percentage variance component.

### Within-genus zooid size

The same species as those analysed for colony size were also assessed for zooid size. Zooid sizes were therefore compared across the K–Pg boundary in two congeneric pairs from the USA and two pairs from Denmark. All selected species have well defined zooids, are abundant and generally show good preservation. Specimens collected in the field were supplemented by material from the collection of Canu and Bassler (1920) at the Smithsonian Institution, Washington D.C. (USNM), including type specimens, the SEM images of which also allowed confirmation of taxonomic identifications. Colonies were selected from two localities within each region to include the influence of local environmental differences (Tables 2, 3).

Colonies were selected for analysis using guidelines adapted from previous studies of zooid size in bryozoans [11], [31]: (1) the colony had to be of reasonable size, with at least ten complete autozooids beyond the primary zone of astogenetic change around the colony origin [32]; (2) the colony had to have encrusted a flat, regular surface, allowing accurate measurement of undistorted zooids; (3) colonies were chosen that occurred on different substrates wherever possible to reduce any substrate-associated biases; and (4) the colony must have been relatively well-preserved and have zooids with well-defined outlines.

For comparisons of zooid size, multiple colonies of the same genus were selected from each locality and stratigraphic horizon to allow for genotypic variation between colonies. Zooids were measured in 30 colonies from the USA (18 Maastrichtian, 12 Danian) and 23 colonies from Denmark (8 Maastrichtian, 15 Danian). These were the only colonies that fulfilled the selection criteria outlined above. SEM images were taken of the selected colonies, either at the Natural History Museum, London (NHMUK), or at the USNM. An area that displayed at least 10 ‘normal’ autozooids, beyond the primary zone of astogenetic change, was selected for scanning.

From each colony, 10 autozooids were randomly selected by numbering each zooid in the SEM image and then randomly selecting 10 of these, excluding: (1) polymorphic zooids such as kenozooids or avicularia; (2) zooids from the primary zone of astogenetic change [32]; and (3) zooids of abnormal size or shape due to physical damage or biotic interactions. In some instances only ovicellate zooids were available for measurement, in which case the ovicells were omitted from the zooidal measurements.

The maximum length and maximum width of each selected zooid was measured using ImageJ software. Zooid area was approximated by multiplying zooid length and width [11]. In total, the length, width, and area of 530 individual zooids were used for this study. Data were normally distributed and a nested ANOVA analysis was therefore conducted, with the addition of intracolonial comparisons to test for size variation between genetically identical individual zooids within each colony and account for measurement error.

### Assemblage level zooid size

A total of 59 species were identified in the assemblage collections, 22 from the USA (20 Maastrichtian, 2 Danian) and 37 from Denmark (25 Maastrichtian, 12 Danian) (Tables 2, 3).

For each species in these assemblages, a well-preserved colony was selected using the guidelines discussed above. If more than one colony within a species was present, the colony with the best preservation was selected for analysis. A light microscope with a graticule was used to measure the length of 10 randomly selected autozooids from each colony, as for the within-lineage study. Only the length was measured in this instance as environmental conditions have been shown to be most influential on this parameter [27], [33], [34] and length is easier to determine accurately under a light microscope than is width.

As before, a nested ANOVA was applied to the zooid length measurements to test for variation within colonies, between colonies, and between stratigraphical intervals.

## Results

### Within-genus colony size

**Figure 3:**
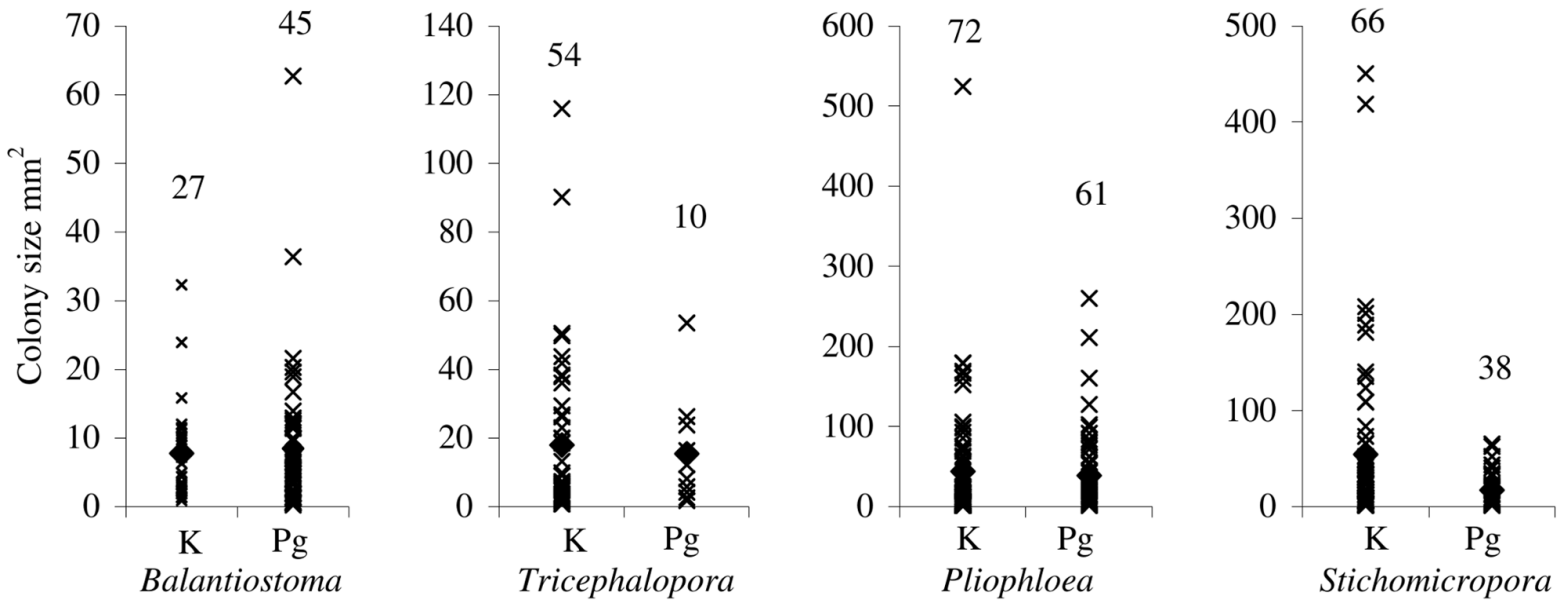
Mean, minimum and maximum colony size for taxa. Colony sizes of taxa analysed from the Maastrichtian Prairie Bluff Chalk/Højerup Member (K) and the Paleogene Clayton Formation/Korsnæb Member (Pg). The total number of colonies measured is indicated above each data point. *p*>0.05 across the K–Pg boundary for each pair.

Mean colony size does not change significantly across the K–Pg boundary for the pairs of species analysed in this study (Table 4, Figure 3, Table S1). *Stichomicropora* showed the greatest variation in colony size, with a decrease of 37.2 mm^2^ (*p* = 0.07). The mean colony sizes of *Tricephalopora* and *Pliophloea subcornuta* also decreased across the boundary, by 2.5 mm^2^ and 5.2 mm^2^ respectively, but these changes are not statistically significant (*p* = 0.26 and *p* = 0.89, respectively). There is also no significant change in colony size between localities for these three genera, suggesting that locality is not a confounding factor (Table 4).

**Table 4:** Nested ANOVA results for colony area.

Congeneric	Mean area (mm^2^)		Min. area		Max. area		F-ratios		% variance		
	(SD)		(mm^2^)		(mm^2^)				component^a^		
	K	Pg	K	Pg	K	Pg	Fm.	Loc.	Fm.	Loc.	Col.
*Balantiostoma nomas*	7.74	8.44	0.84	0.23	32.27	62.39	0.35	7.69***	0	35.21	64.79
	(7.24)	(10.20)									
*Tricephalopora larwoodi*	17.87	15.39	0.72	1.74	115.89	53.48	1.35	0.56	3.07	0	96.93
	(23.85)	(15.89)									
*Pliophloea subcornuta*	43.76	38.61	0.27	1.25	524.07	259.59	0.02	2.16	0	5.71	94.29
	(74.82)	(50.06)									
*Stichomicropora*	54.24	17.07	0.57	0.91	54.24	17.07	4.27	0.50	5.55	0	94.45
	(84.59)	(17.61)									

* = *p*<0.05; ** = *p*<0.01; *** = *p*<0.001.

a Negative estimates of variance components associated with non-significant F-ratios were assigned zero values in keeping with standard practice [26].

The mean colony area of *Balantiostoma* increased across the K–Pg boundary by 0.7 mm^2^, but again this change is not significant (*p* = 0.60). There is, however, significant variation between localities for all *Balantiostoma nomas* colonies (*p*<0.05), which contributes to 35% of the total variance for this species.

Maastrichtian colonies exhibit greater maximum sizes for three of the pairs studied (Figure 3), with maximum colony size decreasing by 50–69% across the K–Pg boundary. However, the opposite trend occurs in *Balantiostoma*, where the largest colony is observed in the Paleocene Clayton Formation, with a 93% increase in maximum colony size when compared to the Prairie Bluff Chalk. Minimum colony size decreases across the K–Pg boundary for *Balantiostoma*, but increases across the boundary for the other taxa studied here (Figure 3).

Colony sizes for these taxa are highly variable, as is shown by their coefficients of variation (CV) (94–171%) and high contributions to the total variation between colonies (65–97%) (Table 4). There is greater variation in the sizes of *Tricephalopora*, *Pliophloea subcornuta* and *Stichomicropora* colonies prior to the boundary, but the variation in *Balantiostoma* colonies is greater in the Danian (CV 121%) than the Maastrichtian (CV 94%).

### Within-genus zooid size

**Figure 4:**
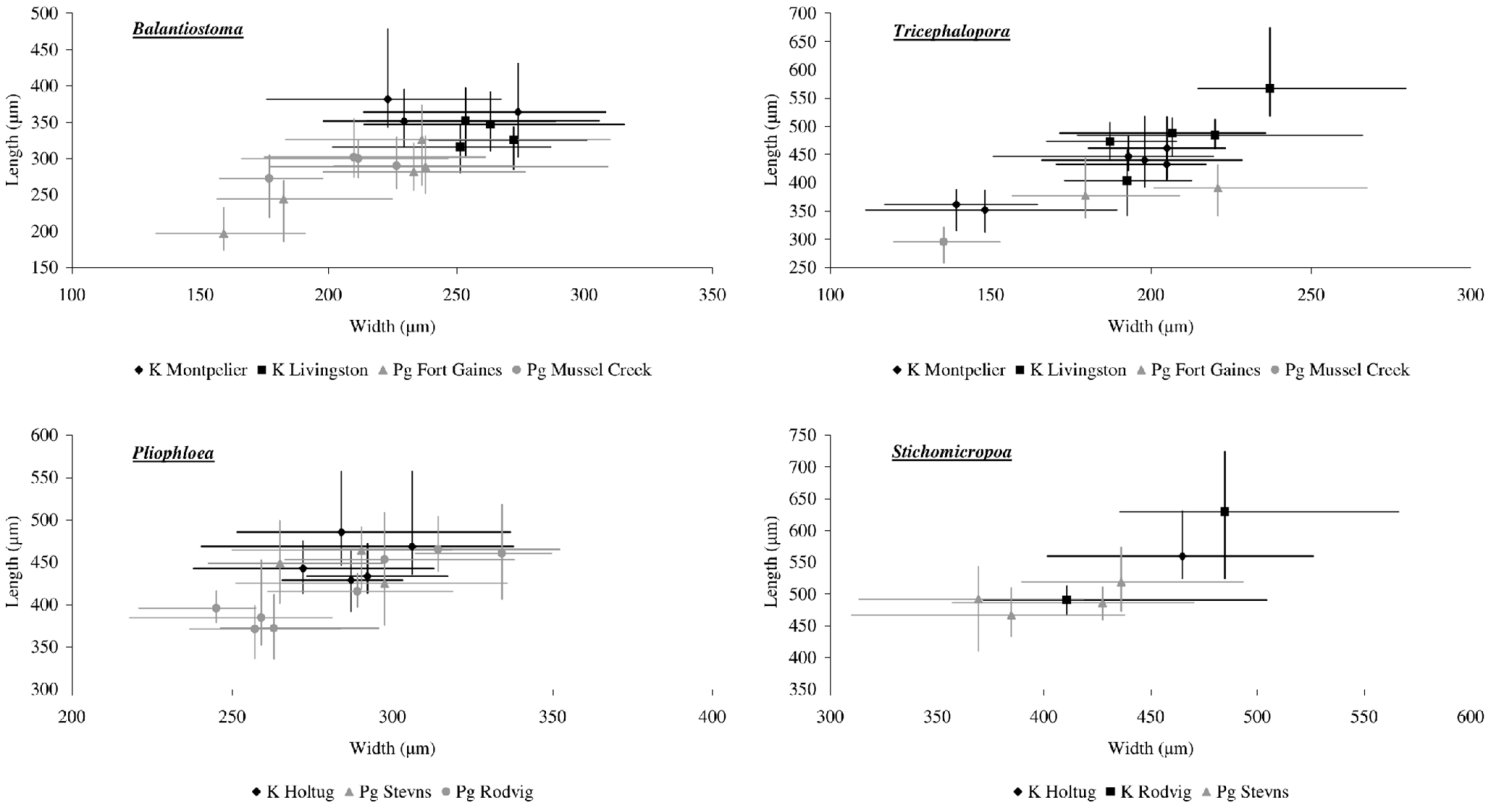
Mean zooid width and length for all colonies. *Balantiostoma*: K = Cretaceous Prairie Bluff Chalk (*Balantiostoma nomas*); Pg = Paleogene Clayton Formation (*B. midwayanica*). ***Tricephalopora:***K = Cretaceous Prairie Bluff Chalk (*Tricephalopora larwoodi*); Pg = Paleogene Clayton Formation (*T. levigatum*). *Pliophloea*:K = Cretaceous Højerup Member; Pg = Paleogene Korsnæb Member. *Stichomicropora*: K = Cretaceous Højerup Member; Pg = Paleogene Korsnæb Member. Error bars show minimum and maximum width and length measured for each colony.

After intra- and inter-colony variation and locality variations were taken into consideration by the nested ANOVA analysis, none of the taxa studied displayed a significant change in zooid length across the K–Pg boundary (Table 5, Figure 4, Table S2). Although mean zooid length declined from the Maastrichtian to the Danian by 29–92 µm (i.e. 6–21%), these decreases were not significant between formations. This variation in size between formations, however, accounted for a significant proportion of the overall variation (45–57%) in all taxa except *Pliophloea subcornuta* (0%) (Table 5). Standard deviations for all taxa in each formation were 32–69 µm around the mean zooid size. There was no consistent directional change in standard deviation, i.e. zooid size was neither more nor less variable on either side of the K–Pg boundary. For all studied taxa, maximum zooid lengths were found in samples from the Maastrichtian, whereas minimum zooid lengths were all recorded from the Danian samples.

**Table 5:** Nested ANOVA results for zooid length.

Congeneric	Mean length (µm) (SD)		F-ratios			% variance component^a^			
	K	Pg	Fm.	Loc.	Col.	Fm.	Loc.	Col.	Zooids
*Balantiostoma nomas*	348 (36)	278 (43)	13.79	1.49	12.63***	57.13	2.90	21.49	18.48
*Tricephalopora larwoodi*	446 (64)	355 (52)	3.98	3.59	29.54***	46.99	19.55	24.78	8.68
*Pliophloea subcornuta*	452 (37)	423 (44)	0.65	1.94	14.47***	0	11.77	50.64	37.59
*Stichomicropora*	560 (69)	491 (32)	n/a^b^	0.00	25.62***	45.12	0	39.03	15.85

* = *p*<0.05; ** = *p*<0.01; *** = *p*<0.001.

a Negative estimates of variance components associated with non-significant F-ratios were assigned zero values in keeping with standard practice [26].

b Denominator of the F-test is zero.

There was no significant difference in zooid length between localities and this factor contributed the least amount of total variation (<20%). There was only minor intracolonial variation in zooid length (9–18%) for taxa other than *Pliophloea subcornuta*. However, between colonies there was significant variation in zooid length for all taxa studied (*p*<0.001) and this accounted for 21–51% of the total variation.

*Balantiostoma* displayed a decrease in zooid width across the K–Pg boundary (*p* = 0.03), with a mean decline of 44 µm (18%) (Figure 4). All other taxa measured showed no significant variation in zooid width between formations, with mean size differences of 15–49 µm (2–11%) (Table 6, Figure 4). Standard deviations remained reasonably constant at 26–50 µm.

**Table 6:** Nested ANOVA results for zooid width.

Congeneric	Mean width (µm) (SD)		F-ratios			% variance component^a^			
	K	Pg	Fm.	Loc.	Col.	Fm.	Loc.	Col.	Zooids
*Balantiostoma nomas*	253	208	24.26*	0.41	8.63***	38.13	0	26.78	35.09
	(33)	(39)							
*Tricephalopora larwoodi*	194	179	0.89	3.51	17.11***	0	32.64	41.56	25.80
	(34)	(40)							
*Pliophloea subcornuta*	288	283	n/a^b^	0.01	12.17***	1.83	0	51.81	46.36
	(27)	(33)							
*Stichomicropora*	454	404	n/a^b^	0.13	10.24***	36.67	0	30.41	32.92
	(50)	(45)							

* = *p*<0.05; ** = *p*<0.01; *** = *p*<0.001.

a Negative estimates of variance components associated with non-significant F-ratios were assigned zero values in keeping with standard practice [26].

b Denominator of the F-test is zero.

Maximum zooid width was observed in the Maastrichtian for all taxa, excluding *Pliophloea subcornuta*, for which a maximum width of 393 µm was observed in the Korsnæb Member compared to a value of 364 µm in the Højerup Member. Similarly, minimum zooid width was always observed in the Danian, except for *Tricephalopora* which had a minimum width of 111 µm in the Prairie Bluff Chalk compared to a value of 120 µm in the Clayton Formation.

As was the case for zooid length, there was no significant variation between localities in zooid width, and *Tricephalopora* is the only genus to contribute to the total variance component for localities (33%). Between 26 and 46% of total variance was intracolonial. Variation between colonies was significant for all species (*p*<0.001) and contributed a higher component of the total variance than that seen for zooid length (27–52%).

Zooid area results are similar to those obtained for zooid length and zooid width, which is unsurprising given that zooid area is a function of these two parameters. *Balantiostoma* was the only taxon to show a decrease in zooid area (*p*<0.001) at the K–Pg, with the average area of zooids declining by 0.03 mm^2^ (33%) from 0.09 mm^2^ to 0.06 mm^2^ (Table 7). The other three taxa showed a decrease in mean area of 0.01–0.06 mm^2^ (7–26%) across the boundary, which was not significant. There is no significant variation between localities for zooid area, but variation between colonies accounts for between 21% and 68% of the total variance.

**Table 7:** Nested ANOVA results for zooid area.

Congeneric	Mean area (mm^2^)(SD)		F-ratios			% variance component^a^			
	K	Pg	Fm.	Loc	Col.	Fm.	Loc.	Col.	Zooids
*Balantiostoma nomas*	0.09	0.06	285.96***	0.06	12.12***	60.16	0.00	20.98	18.86
	(0.01)	(0.02)							
*Tricephalopora larwoodi*	0.09	0.06	2.03	3.34	35.31***	23.52	27.08	38.25	11.15
	(0.02)	(0.02)							
*Pliophloea subcornuta*	0.01	0.01	1.23	0.46	22.86***	1.06	0.00	67.88	31.05
	(0.01)	(0.02)							
*Stichomicropora*	0.03	0.02	n/a^b^	0.02	61.62***	48.89	0.00	38.53	12.58
	(0.05)	(0.03)							

*  = *p*<0.05; **  = *p*<0.01; ***  = *p*<0.001.

a Negative estimates of variance components associated with non-significant F-ratios were assigned zero values in keeping with standard practice [26].

b Denominator of the F-test is zero.

### Assemblage level zooid size

**Figure 5:**
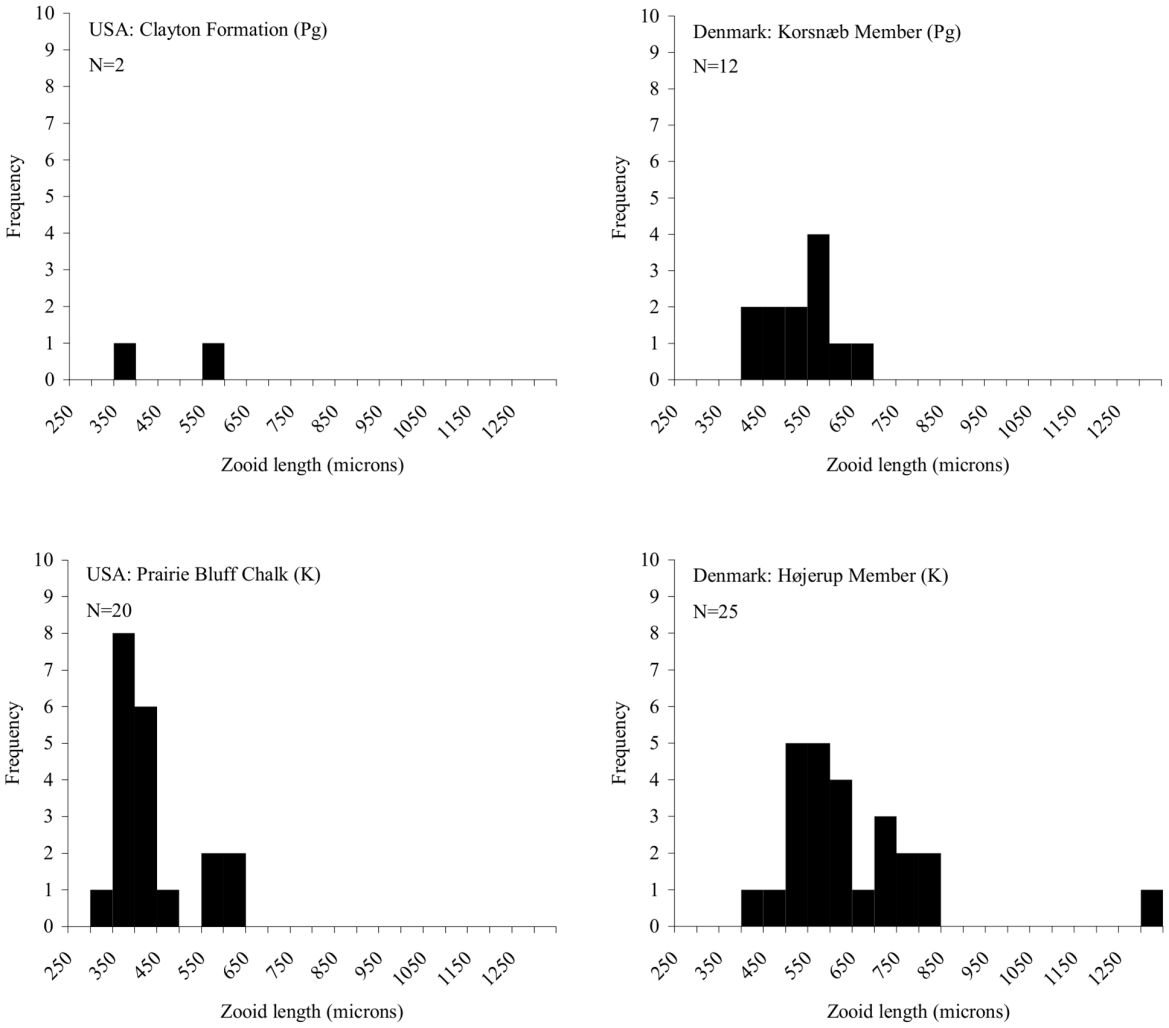
Mean zooid length of all species present in the studied samples. Histograms showing the lengths of zooids from the Maastrichtian Prairie Bluff Chalk/Højerup Member (K) and the Paleogene Clayton Formation/Korsnæb Member (Pg). The total number of species is indicated. *p*>0.05 across the K–Pg boundary in the USA and Denmark in a comparison of overall bryozoan zooid size.

There is no significant change in the length of bryozoan zooids across the K–Pg boundary for species in assemblages from the USA or Denmark (Figure 5, Table S3). In the USA, the overall mean zooid size for bryozoans actually increased by 46 µm across the K–Pg boundary, but this is not statistically significant (*p* = 0.5). In Denmark, overall mean zooid size decreased by 109 µm (*p* = 0.05, which is the level of significance). There is also significant variation in zooid sizes between different species (colonies) within each formation (81%) (Table 8).

**Table 8:** Nested ANOVA results for zooid length of all species.

Region	Mean length (µm)(SD)		F-ratios		% variance component^a^		
	K	Pg	Fm.	Col.	Fm.	Col.	Zooids
USA	386 (87)	432 (95)	0.48	129.15***	11.78	81.86	6.36
Denmark	599 (181)	490 (83)	4.08	219.18***	15.44	80.86	3.81

* = *p*<0.05; ** = *p*<0.01; *** = *p*<0.001.

In both regions, the longest zooids occur in Maastrichtian formations, with *Conopeum spissamentum* in the Prairie Bluff Chalk (550 µm) and *Ellisina simplex* in the Højerup Member (1299 µm) having the greatest mean zooid lengths. *Ellisina simplex* also exhibits the maximum zooid length observed overall in the Højerup Member (1408 µm), but this taxon is not observed in the Korsnæb Member assemblage studied here, where the maximum zooid length is 693 µm and occurs in the measured “*Membranipora*” sp. colony. In the USA, the maximum zooid length in the Prairie Bluff Chalk is observed in a *Tricephalopora larwoodi* colony measured for this analysis (627 µm). The longest zooid present in the Clayton Formation is also in a *Tricephalopora* colony (576 µm). Maximum zooid length therefore appears to decrease across the K–Pg boundary in both regions. The shortest zooid lengths are also observed in the Maastrichtian assemblage for the USA (*Dionella rinsbergi*: 224 µm) and Denmark (*Cryptostomella* sp.: 310 µm). The minimum zooid lengths in the Danian of the USA (*Balantiostoma midwayanica*: 320 µm) and Denmark (*Pliophloea subcornuta*: 347 µm) are longer than the minimum zooid lengths measured in the corresponding Maastrichtian assemblages (Figure 5), although this may be an artefact of the smaller number of species measured in the Danian.

The only species that crosses the boundary and exhibits a size increase is *Cryptostomella* sp. in Denmark. The remaining 6 species that are present in the assemblages on either side of the boundary appear to decrease in zooid length or maintain a relatively constant zooid length across the K–Pg boundary.

## Discussion

There is no significant difference in either zooid size or colony size for any of the analysed cheilostome bryozoan species pairs across the K–Pg boundary, with the exception of *Balantiostoma* which exhibits a significant decease in zooid width and zooid area (but not zooid length) across the boundary, and *Stichomicropora* which decreases slightly in colony size across the boundary. Zooid size in species of *Tricephalopora*, *Pliophloea* and *Stichomicropora* do not vary significantly between formations, nor does colony size in *Balantiostoma*, *Tricephalopora* and *Pliophloea*. In a more general sense, zooid size does not change significantly across the K–Pg boundary when all bryozoan species are considered together in the USA, although there is a slight decrease in zooid size in the Danish assemblage studied here. Variations between localities do not appear to affect colony or zooid size, but there are highly significant between colony variations in zooid size for all taxa in all zooid parameters measured.

### Colony size across the K–Pg boundary

Mean colony size in the majority of cheilostome bryozoans analysed here does not change significantly across the K–Pg boundary, although the mean size of *Stichomicropora* colonies does decrease slightly. This lack of change contrasts with previously reported decreases in colony size at other biotic crises among graptolites [1] and corals [35] and implies that not all colonial organisms exhibit a Lilliput effect.

Colony size in living bryozoans has been shown to be dependent on both quantity and quality of food supply [36]–[38]. As suspension feeders, it was expected that bryozoans would exhibit a decrease in size following the hypothesised primary productivity crash of the K–Pg event. In free-living bryozoans, colony size has been used as a measure of the favourability of environmental conditions based on the fact that larger colonies have a greater number of feeding and reproductive zooids [18]. A pre-extinction decrease in bryozoan colony size in *Lunulites pseudocretacea* in the late Maastrichtian of Nye Kløv, Denmark was attributed to low primary productivity levels [18]. In contrast, no evidence for a significant decrease in colony size across the K–Pg boundary itself at Stevns Klint is apparent in the *Pliophloea subcornuta* colonies studied here, although *Stichomicropora* does exhibit a slight but insignificant decrease in colony size.

Colony size is a function of zooid size and the number of constituent zooids. In the majority of bryozoan species studied here the size of the zooids remained more or less constant across the K–Pg boundary, indicating that there must also be limited variation in the number of zooids per colony for *Tricephalopora* and *Pliophloea subcornuta*. The decrease observed in zooid size (area) in *Balantiostoma*, however, implies that the average number of zooids per colony must increase in order to maintain the constant average colony size observed for this genus. This suggests that, for *Balantiostoma*, the number and size of zooids are more plastic than is total colony area.

The size of the substrates encrusted by bryozoans may place an upper bound on colony size. It is therefore necessary to determine whether colony size responds indirectly to environmental variations or instead adapts to substrate size, which itself may vary according to environmental factors. Although anecdotal observations show a decrease in substrate size across the K–Pg boundary for the material analysed here, and echinoid size has previously been shown to decrease across the K–Pg boundary [13], the limited size response observed in the colonies studied here suggests that colony size does not simply scale to substrate size.

There is, however, a clear decrease in the maximum size of colonies across the K–Pg boundary, for all studied taxa apart from *Balantiostoma*. This apparent decline in maximum colony size indicates that, whilst the mass extinction appears to have had negligible influence on the mean size of these colonies, the extremely large colony sizes reached in the Maastrictian were not attained in the Danian.

A possible reason for the lack of mean colony size decrease found in this study is that sampling across the K–Pg boundary may have been over a timescale that extended beyond the duration of any Lilliput effect, which is in the order of a few hundred thousand years [1]. The duration of the size response has important implications as it can help to determine whether Lilliput effects are short-term ecological responses or represent long-term resettings of evolutionary history [3]. The samples used in this study were not collected from sediments deposited immediately post-extinction (Figure 1) and may have failed to detect a short-term Lilliput effect: size may have returned back to ‘normal’ pre-extinction level by the time of the Paleocene bryozoans used for this study. In particular, the Danish specimens come from the Korsnæb Member as too few bryozoans are present in the Cerithium Limestone immediately above the K–Pg boundary. Likewise, a depositional hiatus underlying the sampling levels for the Clayton Formation bryozoans from the USA that were used in this study is likely [39].

### Zooid size across the K–Pg boundary

#### Decrease in zooid width and area of Balantiostoma across the K–Pg boundary

Of the genera studied here, only *Balantiostoma* showed significant changes in size across the K–Pg boundary, with a decrease in both zooid width and area. To interpret the cause of this size decrease, it is necessary to consider the standard phenotypic responses of bryozoan zooids to unfavourable conditions [1]. A reduction in primary productivity has been hypothesised as the cause for small organism size following the K–Pg event [13], [17], although an array of other environmental stresses, such as greenhouse warming, shallowing seas and the restriction of basins, as well as volcanic activity during the late Maastrichtian, have been cited as possible causes for a trend towards small-sized foraminifera [40]. Alternative environmental factors suggested to induce Lilliput effects at mass extinctions elsewhere in the geological record include anoxia, and changes in temperature and salinity levels [2].

Environmental influences on zooid size in living cheilostome bryozoans have been investigated using laboratory and field experiments [31], [41]. Temperature is often identified as the most important single environmental influence on zooid size, with an inverse relationship between size and temperature both within colonies and within species [27], [33], [37], [41]–[47]. This relationship is believed to be a taxon-independent function of metabolism at different temperatures [44]. Therefore, the zooid size-temperature trend should not have altered through geological time [11]. Although there is some evidence that a zooid size-temperature relationship may occur between congeneric species [48], applying this relationship to changes in zooid size between related species across the K–Pg is more difficult to justify given our current state of knowledge. For example, it seems unlikely that only *Balantiostoma* among the genera studied would have experienced temperature-related zooid size reduction, especially as the studied colonies are from the same localities as *Tricephalopora* colonies that showed no change in zooid size.

Alternative environmental parameters that have been shown to influence zooid size include salinity and oxygen [37]. Based on studies of other groups, the likeliest driver of any zooid decrease at the K–Pg boundary is the decline in primary productivity hypothesised to have occurred at this time [12]. However, contradictory results have been published on the effect of food quality and quantity on zooid size in modern bryozoans [37], [42], [45]. Since food supply has sometimes been shown to have no influence on zooid size, it has been suggested that its impact is slight and overprinted by temperature [46]. However, a positive correlation between zooid size and both food quality and quantity in laboratory experiments on *Electra pilosa* has been observed [38]. Possible explanations for mixed results in food studies on zooid size include different methodologies [38], the fact that laboratory experiments do not reflect the true complexities of the natural world [45], and a non-uniform response of different bryozoan species to changes in food supply [49]. The implication of zooid size decrease only occurring in *Balantiostoma* may be that not all bryozoan genera were similarly affected by the hypothesised primary productivity decline across the K–Pg boundary.

The lack of change in zooid length in *Balantiostoma* at the K–Pg underlines the importance of obtaining multiple measurements of body size and understanding the implications that such parameters may have on the physiology and ecology of the studied organisms. In *Balantiostoma*, the variation in zooid width but not length contrasts with previous bryozoan zooid size studies that have found zooid length to be the most sensitive size parameter to environmental changes [27], [33], [34]. It has been suggested that zooid width is determined mainly by the position of the zooid in the colony, particularly with respect to row bifurcations, whereas zooid length is more dependent on environmental influences [34], [37]. Further knowledge of the relationship between these size parameters would help to understand why most change in *Balantiostoma* is in width rather than length. At the assemblage-level, additional analysis of trends in zooid width might therefore also aid further insight into bryozoan size response.

#### Lack of consistent zooid size change

The results of this study contradict the Lilliput hypothesis, which predicts significant size decrease across the K–Pg boundary in all species. None of the four analysed species show significant changes in zooid length at this boundary, and there is also no significant change in zooid width or area for three of these species. Furthermore, a more general analysis of zooid size in assemblages does not show a significant trend to smaller size after the K–Pg mass extinction in the USA, in contrast to previous whole assemblage size analyses, which constitute the majority of Lilliput studies [2], [50], although a slight decrease in zooid size was detected in the Danish assemblages.

However, size change at mass extinction horizons is likely to be complex and it cannot be expected that all clades will respond in the same manner, or that all mass extinctions will have the same effects [10]. The definition of the Lilliput effect allows for more variety in the response of organisms than is often supposed [8]; in Urbanek's original study of the Lilliput effect [1], size decrease was not consistent across all species, and studies of within-lineage size across mass extinction events have since obtained mixed results (Table 1). Furthermore, the difference in size trends between *Balantiostoma* and the three other bryozoan genera studied here demonstrates that even quite closely related taxa do not always respond identically [8].

As with colony size, it is possible that the timescale of any Lilliput effect on bryozoan zooid size was too short to be detected in this study. It is also possible that zooid sizes prior to the K–Pg event were smaller than ‘normal’. Pre-extinction dwarfing has previously been observed in other taxa [51], [52], and an observed shift to smaller bryozoan zooid size prior to the K–Pg boundary in Denmark was attributed to a change in temperature [18]. These results imply that ‘unfavourable’ environmental conditions affected zooid size leading up to the mass extinction, potentially ‘re-setting’ size trends prior to the K–Pg boundary rather than across the K–Pg boundary itself. This trend corresponds with a suggestion that the biotic responses to the K–Pg event were not instantaneous and that environmental perturbations predated the K–Pg boundary [53]. However, the size decrease observed in *Balantiostoma* indicates that at least one species experienced a change on the timescale used in this study and the decline in assemblage-level zooid size in Denmark also suggests an appropriate timescale was analysed. It is unclear, therefore, whether these results indicate lack of a size response, or different timings of size responses between species. Analysis of additional colonies extending a further time distance from the boundary are therefore necessary in order to establish the true extent and influence of the K–Pg event on bryozoan zooid size.

#### Genotypic variation

In all parameters measured for all species studied, there are highly significant variations in zooid size between colonies, underlying similarities found at the formation level. These variations may be inferred as genotypic in origin, which is known to be responsible for a large amount of zooid size variation observed within species of living bryozoans [37], [38], [42]. Therefore, caution must be taken when inferring environmental factors influencing bryozoan zooid size [48]; mean zooid size can often vary more between genotypes of the same species than over time or between environments [46]. Genotypic effects can be expected to be even greater among closely related, congeneric species, like those analysed in the current study. However, the nature of the nested ANOVA analysis demonstrates that the differences and similarities at the formation level (i.e. across the K–Pg boundary) occur despite size variation within and between colonies from each formation.

### Biogeographical and regional trends

The two regions from which the bryozoans were collected contrast markedly in their depositional environments, latitude, distance from the Chicxulub impact size, and types of hard substrates available. The fact that both show a similar lack of change in zooid size across the boundary indicates that such stasis is more likely to be a global than a regional pattern [20]. If localised variations had been influential on bryozoan size, greater variation in size trends between the two regions would be expected. For example, it has previously been shown that colonies of a modern cheilostome species living on different substrate types differ in size [34], but a similar pattern is not evident in the current study.

For within-lineage analyses, excluding *Balantiostoma* zooid size and *Stichomicropora* colony size, there is no significant difference between zooid or colony sizes of any other studied species at the locality level. This implies that size patterns override local differences in environments, with no regional variation detected in this study. Additionally, analysis of two species from the USA and two species from Denmark shows a similar lack of zooid size response in these biogeographical regions. The only genus to display a significant decrease in zooid size is *Balantiostoma*, but as *Tricephalopora* colonies from the same localities failed to show a parallel trend, this indicates that the change was taxon-specific. There is also a difference in zooid size trends between regions at the assemblage level, with Danish cheilostome zooid sizes decreasing slightly across the boundary while the USA sizes remained static.

## Conclusions

1. Bryozoan colony size and zooid size do not change significantly across the K–Pg boundary in two widely separated biogeographical regions, Denmark and the southeastern USA. The influence of locality on colony size is variable and is significant only for *Balantiostoma* from the SE USA among the four genera studied.
2. Although *Stichomicropora* did show a slight decrease in colony size, the size of colonies for the three other genera studied here remained static across the K–Pg boundary, contradicting the predicted decline reflecting a Lilliput effect and some previous studies of colony size change in other colonial groups across mass extinctions [1], [34].
3. Zooid size generally remained stable in assemblages across the K–Pg boundary in the USA, which is in contrast to size decreases observed at the assemblage-level in solitary organisms in other Lilliput studies. In Denmark, however, a slight decline in zooid size was observed.
4. Zooid size change across the K–Pg boundary in pairs of congeneric species varied according to species, which is consistent with the original Lilliput study of graptolites [1] and more recent analyses at lower taxonomic levels [8]. Three out of the four studied genera showed no significant change in zooid length, width, or area across the K–Pg boundary, whereas zooid width and area were found to decrease in *Balantiostoma*.
5. The slight and non-universal size reduction observed in the bryozoans studied here at both the colony- and zooid-levels across the K–Pg boundary shows that these colonial animals do not exhibit a classic and predictable Lilliput effect, despite the fact that various studies of present-day bryozoans have shown that size is responsive to localised environmental changes.
6. The possibility that size change in bryozoans across the K–Pg boundary occurred over a shorter interval than sampled in this study cannot be ruled out.
7. This caveat apart, the lack of size change in the majority of bryozoans studied here at the K–Pg mass extinction indicates that a universal Lilliput effect occurring across all taxa at all mass extinction events cannot be supported. Instead, it seems probable that different organisms have responded differently in terms of body size to different mass extinctions.

## Supporting Information

Table S1 **Colony sizes for within-genus analysis.**
(XLS)

Table S2 **Zooid sizes for within-genus analysis.**
(XLS)

Table S3 **Measurements of zooid length for assemblage level analysis.**
(XLS)
